# Research progress on the classification and clinical response strategies for “complex ductoscopy operating procedure”

**DOI:** 10.3389/fonc.2026.1794764

**Published:** 2026-08-11

**Authors:** Jipeng Zheng, Yibei Tang, Yang Gao, Yihan Sun, Gang Li, Xiang Fei, Xuanyu Chen, Wanli Zheng, Liangliang Zhu, Jingyao Yu, Yu Zhou, Yaqin Wu, Chaojie Zhang, Xingai Ju, Dongxiao Zhang, Jianchun Cui

**Affiliations:** 1 Department of General Medicine, People’s Hospital of China Medical University (People’s Hospital of Liaoning Province), Shenyang, China; 2 Department of Thyroid and Breast Surgery, People’s Hospital of China Medical University (People’s Hospital of Liaoning Province), Shenyang, China; 3 Department of Cardiology, People’s Hospital of China Medical University (People’s Hospital of Liaoning Province), Shenyang, China; 4 Dalian Medical University, Dalian, China; 5 Department of Emergency Medicine, People’s Hospital of China Medical University (People's Hospital of Liaoning Province), Shenyang, China; 6 Department of Galactophore, Beijing Hospital of Traditional Chinese Medicine, Capital Medical University, Beijing, China; 7 Department of Breast and Thyroid Surgery, Hunan Provincial People’s Hospital, Changsha, China

**Keywords:** bloody nipple discharge, ductal stenosis, ductoscopy, meta-analysis, nipple deformation, no spontaneous nipple discharge

## Abstract

**Objective:**

To systematically review the literature on ductoscopy-related operative failures, establish a classification system for “Complex Ductoscopy Operating Procedure (CDOP)”, and propose standardized clinical response strategies.

**Methods:**

A comprehensive search of PubMed, Embase, Web of Science, Scopus, and Cochrane Library was conducted for publications from January 1991 to July 2026. Causes of ductoscopy failure were extracted, categorized, and quantitatively summarized. The methodological quality of included studies was evaluated. Based on literature evidence and accumulated clinical experience, a standard CDOP-PFN (Passing, Finding, Non-discharge) classification system and stepwise clinical management framework were developed.

**Results:**

From 25 included studies (2,853 procedures), meta-analysis showed a pooled failure rate of 10.0% (95%CI: 6.8%–13.7%; 95% PI: 0.0%–31.4%) with high heterogeneity (I² = 87.1%). Primary causes were ductal perforation (22.5%), ductal stenosis (21.9%), and nipple deformity/retraction (12.1%). Failures were stratified into three major types (PFN): (i) presence of nipple discharge with inability to access the ductal system via ductoscopy (type P, passing); (ii) bloody discharge with failure to identify intraductal lesions (type F, finding); and (iii) absence of spontaneous discharge but ultrasonographic evidence of ductal dilatation and intraductal lesions (type N, non-discharge). For each type, a tiered strategy was formulated, incorporating pre-procedural evaluation, duct entry optimization, image-guided localization, troubleshooting, and surgical approaches when needed.

**Conclusion:**

The proposed CDOP-PFN classification and management framework standardizes the recognition and handling of complex ductoscopy operating scenarios. These strategies may reduce dependency on individual operator experience, improve procedural standardization and safety, enhance diagnostic yield for intraductal lesions, and advance minimally invasive breast disease management.

## 1 Research background

Ductoscopy, an endoscopic technique adapted from mature endoscopic platforms, has evolved considerably over the past three decades and is now widely implemented worldwide for the diagnosis and treatment of pathological nipple discharge (PND) (1). With progressive miniaturization of endoscopes and refinement of operation techniques, ductoscopy has demonstrated increasing clinical utility, especially in the early detection of micro-intraductal lesions.

The “Clinical Practice Guidelines for Ductoscopy (2024 Edition)” and “Expert Consensus on Clinical Diagnosis and Treatment of Ductoscopy (2022 Edition)” (1, 2) issued in China have promoted standardized protocols for ductoscopy process, showing its clinical implementation. Despite these advances, procedure-related failure remains relatively common in clinical practice. Clinical difficulties are frequently observed in three recurring scenarios: (1) Spontaneous nipple discharge present, but the ductoscope fails to enter the target ductal lumen (type P); (2) Bloody nipple discharge present, but no intraductal lesions are identified endoscopically (type F); (3) No spontaneous nipple discharge, yet breast ultrasonography suggests ductal dilatation and intraduct space-occupying lesions (type N).

To address these complex procedural challenges, our team first proposed the concept of the “Complex Ductoscopy Operating Procedure (CDOP)” during the 2nd National Forum of Directors of Breast Departments of Integrated Traditional Chinese and Western Medicine in 2018. Since then, we have initiated a systematic research effort to establish a structured classification system and corresponding clinical management strategies for CDOP.

This study aims to develop an literature-and experience-derived classification framework for complex ductoscopy, integrate findings from literature and clinical practice, and formulate standardized operation procedures to improve the success rate and the diagnostic yield of ductoscopy in the evaluation of intraductal breast disease.

## 2 Basis for the classification of CDOP

**Figure 1:**
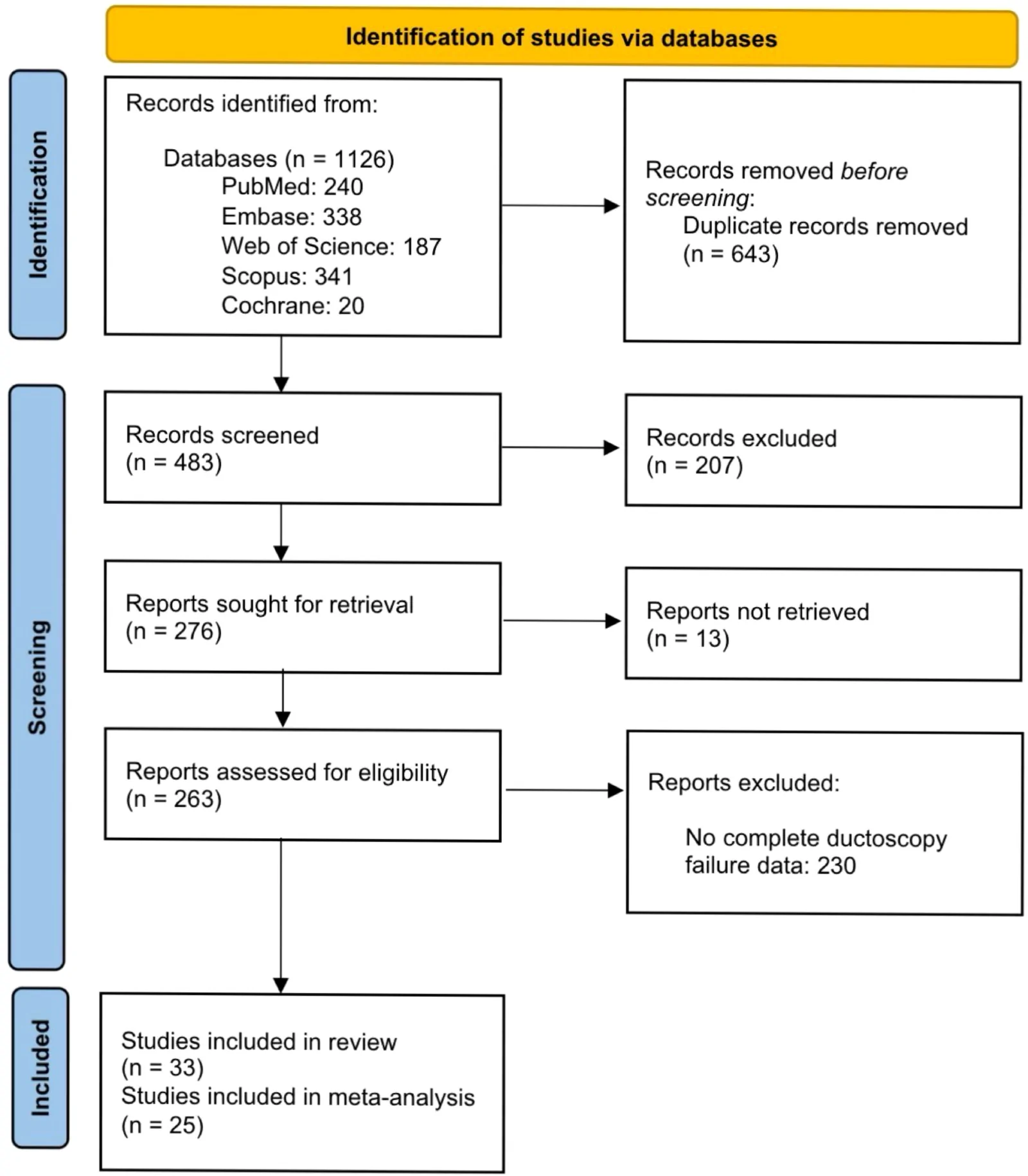
PRISMA 2020 flow diagram summarizing the study selection process.

A systematic literature search was conducted in five electronic databases—PubMed, Embase, Web of Science, Scopus, and Cochrane Library—to identify publications from January 1991 to July 2026. The search strategy employed a comprehensive set of nine synonymous terms related to ductoscopy, including “ductoscopy,” “mammary ductoscopy,” “breast ductoscopy,” “fiberoptic ductoscopy,” “fibreoptic ductoscopy,” “galactoscopy,” “nipple discharge endoscopy,” “duct endoscopy,” and “intraductal endoscopy,” combined using the Boolean operator OR. The initial search yielded 1,126 records across the five databases (PubMed: 240; Embase: 338; Web of Science: 187; Scopus: 341; Cochrane: 20). After removing duplicate records (n=643) and excluding non-original article types such as conference abstracts, reviews, and commentaries (n=207), 276 reports were sought for retrieval, of which 13 could not be obtained. The remaining 263 reports were assessed for full-text eligibility, and 230 were excluded due to absence of extractable ductoscopy failure data. A total of 33 studies were included in the qualitative synthesis, of which eight reports from overlapping patient cohorts were excluded, leaving 25 independent studies for the final meta-analysis (**Figure 1**).

The methodological quality of the 33 included studies was independently assessed using appropriate tools based on study design: the Newcastle-Ottawa Scale (NOS) for cohort studies, the Joanna Briggs Institute (JBI) Critical Appraisal Checklist for case series, QUADAS-2 for diagnostic studies, and the Cochrane Risk of Bias 2.0 (RoB 2) tool for randomized controlled trials. Of the 33 reviewed articles, 32 (97.0%) were rated as Good quality and 1 (3.0%) as Fair; no studies were classified as Poor. A detailed quality assessment is provided in **Supplementary File 1**. All included studies reported explicit ductoscopy failure events with identifiable failure mechanisms and case counts. This evidence base provides the scientific foundation for establishing CDOP classification criteria and analyzing the main causes of ductoscopy failure.

### 2.1 Inclusion criteria

- Study Design

Eligible studies included prospective or retrospective cohort studies, case series, case reports, diagnostic studies, randomized controlled trials, and technical analysis reports that clearly documented intraoperative ductoscopy failure events. Studies were required to provide explicit descriptions of specific failure etiologies—such as procedural technical limitations, anatomical abnormalities, device-related issues, or intraoperative complications—and supply either quantitative failure counts or qualitative classifications of failure modes.

- Study Population

Studies were required to involve patients undergoing ductoscopy for diagnostic or therapeutic indication, irrespective of age, sex, or underlying pathology. A minimum sample size of ≥10 patients was required for cohort-based research. Case reports and small case series (<10 patients) were included only when they provided comprehensive, clinically informative and clearly delineated descriptions of procedural failure mechanisms.

- Language and Timeframe

Only peer-reviewed publications published between January 1, 1991, and July 12, 2026, were included, with no language restrictions applied.

- Data Completeness and Clarity

Included studies must have differentiated primary intraoperative failure causes (e.g., entry into a “false passage,” ductal perforation, ductal stenosis, and equipment malfunction) from procedural consequences (e.g., premature termination of examination, failure to cannulate the duct, and inability to visualize or reach lesions). Sufficient granularity in data reporting was required to support systematic extraction and synthesis.

### 2.2 Exclusion criteria

- Study Design Limitations

Studies without original data—such as narrative reviews, expert commentaries, editorials, opinions, and conference abstracts—were excluded. Animal studies, cadaveric or phantom model studies, and *in vitro* technical experiments were also excluded. In addition, studies combining ductoscopy with other modalities (e.g., galactography) were excluded when ductoscopy-specific results could not be independently extracted.

- Insufficient Data Reporting

Studies were excluded if they did not provide adequate procedural failure information to enable reliable data extraction and synthesis. Specifically, exclusion applied to studies that: (i) did not clearly specify the etiologies or mechanisms of ductoscopy failure; (ii) described procedural failure in vague, non-quantifiable terms (e.g., “some failures occurred,” “a few unsuccessful cases”); (iii) failed to report explicit numbers of failed procedures or present sufficient extractable data to characterize failure events.

- Duplicate Publications

When duplicate or overlapping datasets from the same patient cohort were published across multiple articles, the most complete and methodologically robust report was retained, and the others were excluded to avoid duplication bias.

- Lack of Direct Relevance to Procedural Failure

Studies focusing on technical device innovation, postoperative outcomes, pathology findings, or diagnostic performance without direct reporting of intraoperative failure events and causes were excluded. Research emphasizing ductoscopy-assisted procedures without independently reported ductoscopy failure metrics was excluded.

### 2.3 Statistical analysis of the causes of ductoscopy operation failures

A total of 2,853 ductoscopy procedures reported in eligible studies from 1991 to 2026 were included in the pooled analysis (**Table 1**). Analyses included calculation of proportions and their 95% confidence intervals (via the Clopper–Pearson exact method), comparison of proportions (using Bonferroni-corrected two-proportion Z-tests), assessment of temporal trends (Cochran–Armitage test), and meta-analysis of the overall failure rate. For the meta-analysis, proportional data were stabilized using the Freeman-Tukey double arcsine transformation, and pooled estimates were calculated using a random-effects model with restricted maximum likelihood estimation (REML). Heterogeneity was quantified using the I² statistic and assessed via Cochran’s Q test. For each failed procedure, only the single most contributory cause was recorded to ensure mutual exclusivity of categories. When aggregating the detailed failure causes from **Table 1** into the five common clinical categories presented in **Table 2**, each failed procedure was assigned to only one category. The classification process was performed independently by two authors (J.Z. and Y.T.), with disagreements resolved by consensus or consultation with a third author (J.C.).

**Table 1:** Summary of ductoscopy failure rates and etiologies in published studies (1991-2026).

No.	Total procedures	Failure cases	Failure rate (%)	Major failure causes (count)	Reference
1	27	1	3.7	① Nipple deformation/Scar (1)	Dooley, 2002 (3)
2*	119	14	11.8	① Equipment-related issues(2); ② Ductal stenosis(5); ③ Lesions or stenotic segments located posterior to the nipple(7)	Dietz et al, 2002 (4, 5)
3	30	11	36.7	① No nipple discharge(11)	Kim, J.A., et al, 2004 (6)
4	68	9	13.2	① Ductal perforation(8); ② Ductal stenosis(1)	Moncrief et al, 2005 (7)
5	50	10	20.0	① Entry into a false passage(10)	Beechey-Newman et al, 2005 (8)
6*	54	4	7.4	① Ductal stenosis(1); ② Ductal perforation(1); ③ Equipment-related issues(2)	Hü nerbein et al, 2006 (9–11)
7	26	3	11.5	① Nipple deformation/Sclerosis(1); ② Impaired view due to methylene blue(1); ③ Duct obstruction(1)	Al Sarakbi et al, 2006 (12)
8	20	2	10.0	① No nipple discharge(1); ② Entry into a false passage(1)	Grunwald et al, 2006 (13)
9	308	28	9.1	① Ductal perforation(21); ② Undetermined(7)	Tondre et al, 2008 (14)
10	66	3	4.5	① Duct obstruction(1); ② Impaired view due to hematoma (1); ③ Equipment-related issues(1)	Simpson, J. S., et al, 2009 (15)
11	25	9	36.0	① Ductal stenosis(9)	Cyr, A. E., et al, 2011 (16)
12	121	2	1.6	① Ductal stenosis(2)	Fisher et al, 2011 (17)
13	59	9	15.3	① Entry into a false passage(4); ② Ductal stenosis(2); ③ Ductal perforation(3)	Khan, S. A., et al, 2011 (18)
14	100	2	2.0	① Ductal stenosis(2)	Albrecht et al, 2013 (19)
15*	456	73	16.0	① Ductal stenosis(33); ② Ductal perforation(25); ③ Nipple deformation/Retraction/Ulceration/Ectopic nipple duct(13); ④ Intolerable pain(2)	Kamali et al, 2014 (20–22)
16	164	16	9.8	① Ductal stenosis(8); ② Difficulty in identifying the ductal orifice(6); ③ Lesions or stenotic segments located posterior to the nipple(2)	Zielinski J, et al, 2015 (23)
17	238	5	2.1	① Nipple deformation/Retraction(5)	Liu M, et al, 2015 (24)
18*	224	10	4.5	① Entry into a false passage(6); ② Difficulty in identifying the ductal orifice(1); ③ No nipple discharge(1); ④ Insufficient depth(2)	Ralf Ohlinger et al, 2020 (25, 26)
19*	213	62	29.1	① Ductal perforation(28); ② Nipple deformation/Retraction/Prior surgery(16); ③ Ductal stenosis(14); ④ Duct obstruction(4)	Filipe, M. D, et al, 2020 (27–29)
20	223	13	5.8	① Stenosis of the ductal orifice(7); ② Duct obstruction(4); ③ No nipple discharge(2)	Yuk-Kwan Chang et al, 2020 (30)
21	34	4	11.8	① Ductal perforation(4)	Gui et al, 2021 (31)
22	112	4	3.6	① Bloody nipple discharge with no identified lesion(4)	Yang et al, 2023 (32)
23*	35	7	20.0	① No nipple discharge(7)	Makineli et al, 2024 (33)
24*	24	3	12.5	① Ductal perforation(3)	Makineli et al, 2024 (34)
25	57	2	3.5	① Stenosis of the ductal orifice(1); ② Ductal perforation(1)	Cabioglu N et al, 2025 (35)
In total	2853	306	10.7		

*Duplicate publications were identified for studies No.2 (two studies), No.6 (three studies), No.15 (three studies), No.18 (two studies) and No.19 (three studies). For each group, the most comprehensive dataset was retained to avoid duplication bias. Data entries for No.23, No.24 and No.25 originated from UMCU but represented distinct cohorts or time periods, were included as separate datasets.

**Table 2:** Distribution of common clinical categories contributing to ductoscopy failure.

Rank	Common clinical categories	Aggregated cases (n)	Proportion (%)*	95% confidence interval (95% CI)	Comparison with ductal stenosis (P-value)
1	Ductal Stenosis	76	24.8	20.1–30.1	Reference
2	Nipple Deformation	36	11.8	8.4–15.9	<0.001
3	No Nipple Discharge	20	6.5	4.0–9.9	<0.001
4	Lesions in Subareolar Main Duct	9	2.9	1.4–5.5	<0.001
5	Bloody Discharge with no Lesion	4	1.3	0.4–3.3	<0.001
	Total (Common Clinical Categories)	145	47.4	41.7–53.2	

Data were aggregated from 25 eligible studies, covering a total of 306 ductoscopy failure cases. *Proportion represents the weighted percentage relative to the total number of failure cases (n=306). 95% confidence intervals (95% CIs) were calculated via the Clopper-Pearson exact method. P-values for pairwise comparisons with ductal stenosis were derived from two-proportion Z-tests with Bonferroni correction for multiple comparisons. The remaining 161 failure cases (52.6%) were attributed to technical failures (perforation and false passage, 98 cases, 32.0%), device-related issues, procedural pain, impaired visualization, undetermined causes, or other miscellaneous factors (63 cases, 20.6%).

**Figure 2:**
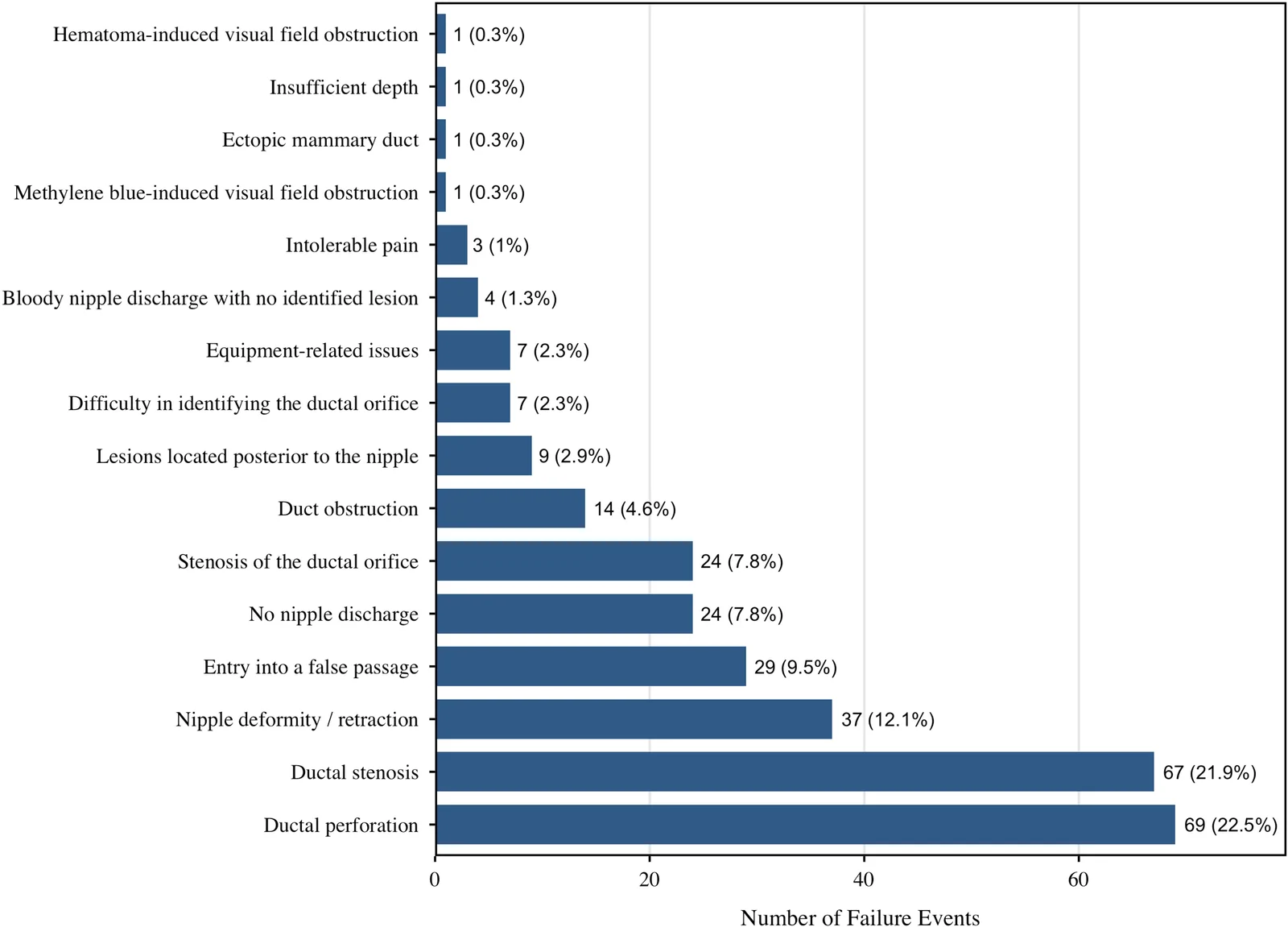
Proportion of causes for ductoscopy failure (e.g., equipment-related issues: 2.3%).

Among these, 306 procedures resulted in failure, yielding an overall failure rate of 10.0% (95%CI: 6.8%–13.7%; 95% prediction interval: 0.0%–31.4%), with substantial heterogeneity across studies (I² = 87.1%, p < 0.0001). Failure etiologies encompassed multiple domains including anatomical complexity, lesion localization challenges, and device-related or operator-related technical limitations (**Figure 2**).

The most frequent causes of ductoscopy failure were ductal perforation (22.5%), ductal stenosis (21.9%), and nipple deformity/retraction (12.1%), accounting for approximately 56.5% of all failures. Additional contributors included entry into a false passage (9.5%), absence of spontaneous nipple discharge (7.8%), stenosis of the ductal orifice (7.8%), and duct obstruction (4.6%). Importantly, ductal perforation and entry into a false passage frequently co-occur during the procedure. Both events reflect intraoperative mechanical misdirection and suboptimal instrument manipulation, typically associated with limited operator experience or inadequate technical proficiency. Accordingly, these causes were considered as “technical failure”, with a combined incidence of 32.0%.

**Figure 3:**
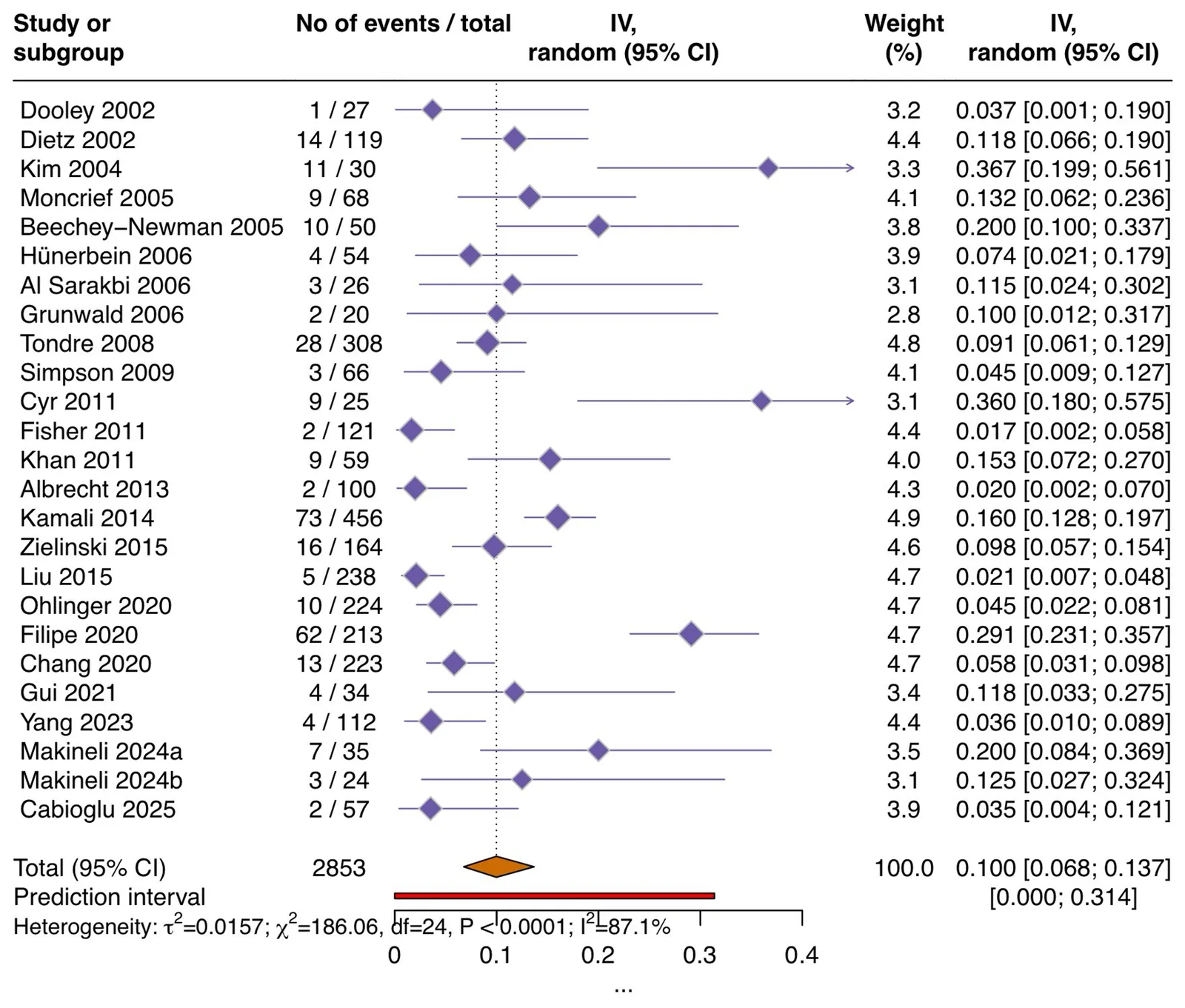
Forest plot of the meta-analysis of ductoscopy failure rates. *Squares represent study-specific failure rates, with size proportional to study weight (inverse variance). Horizontal lines indicate 95% confidence intervals (CIs). The diamond at the bottom represents the pooled estimate using a random-effects model with the Freeman-Tukey double arcsine transformation and restricted maximum likelihood estimation (REML). The width of the diamond indicates the 95% CI. Heterogeneity: I² = 87.1%, P < 0.0001; τ² = 0.0157. The 95% prediction interval (PI) is shown as a dashed line extending from the pooled estimate.

**Figure 4:**
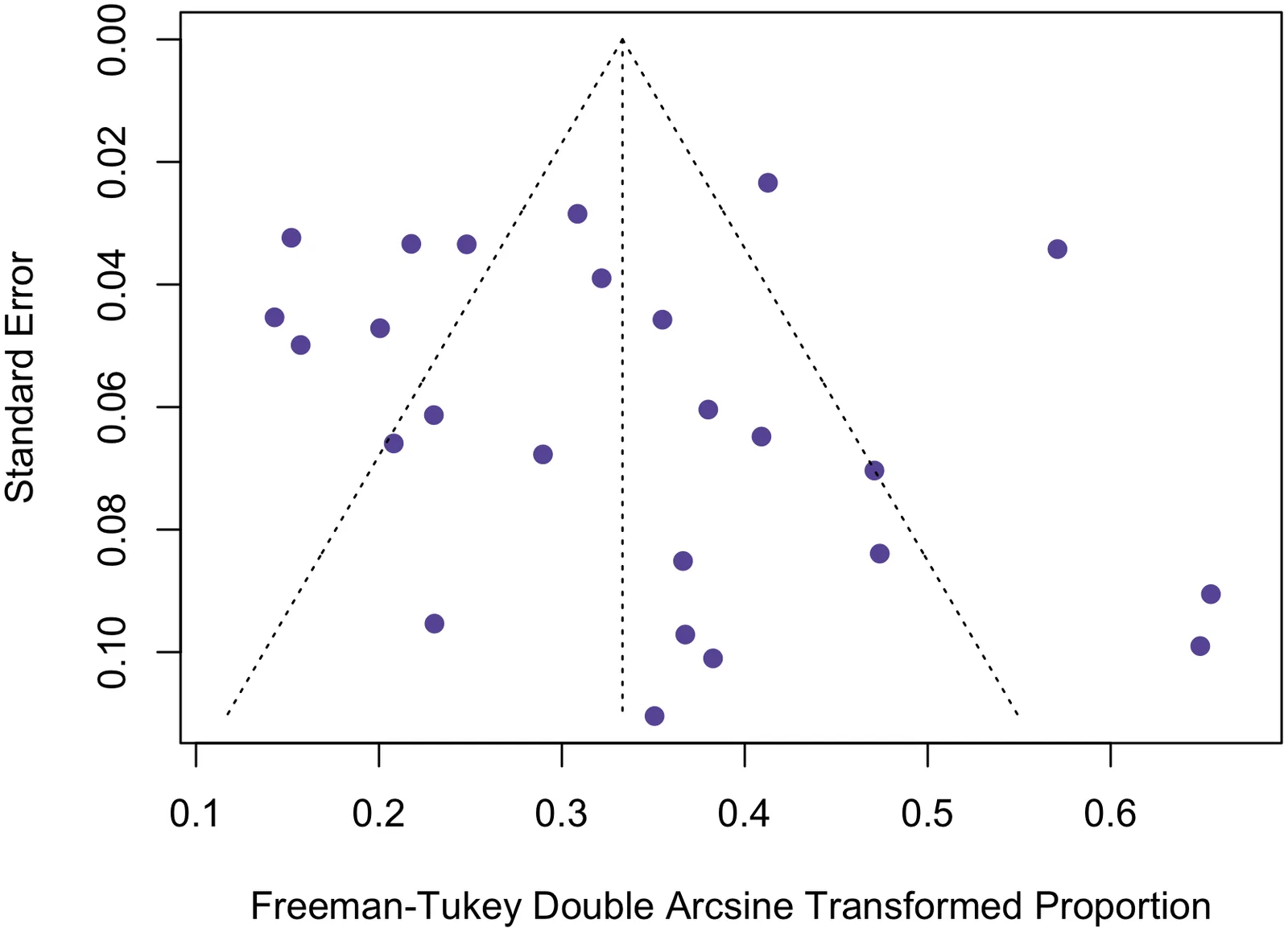
Funnel plot for assessment of publication bias. *Each point represents an individual study included in the meta-analysis. The vertical dashed line indicates the pooled estimate (Freeman-Tukey double arcsine transformed proportion), and the diagonal dashed lines represent the pseudo-95% confidence limits. The funnel plot appeared reasonably symmetrical, and Egger's regression test did not suggest significant publication bias (intercept = 0.2897, P = 0.6164).

To integrate the evidence from these heterogeneous studies, we performed a meta-analysis to estimate the overall ductoscopy failure rate. Using a random-effects model with the Freeman-Tukey double arcsine transformation and restricted maximum likelihood estimation (REML), the pooled failure rate was 10.0% (95% CI: 6.8%–13.7%), with a 95% prediction interval ranging from 0.0% to 31.4%, indicating considerable variability in expected failure rates across different clinical settings (**Figure 3**). Substantial heterogeneity was observed across studies (I² = 87.1%, P < 0.0001; τ² = 0.0157), supporting the use of a random-effects model and justifying the stratification of studies for subgroup analyses. The high heterogeneity likely reflects variations in technical eras, operator expertise, and patient selection criteria among the included studies. Publication bias was further assessed using funnel plot inspection and Egger’s regression test. As shown in **Figure 4**, the funnel plot appeared reasonably symmetrical. Egger’s regression test did not suggest significant publication bias (intercept = 0.2897, P = 0.6164).

To explore potential sources of the substantial heterogeneity observed in the meta-analysis, we performed subgroup analyses stratified by geographic region, study design, and time period. Stratified by geographic region, the highest pooled failure rate was observed in the Netherlands (23%, 95% CI: 14%–33%), followed by the UK (15%, 95% CI: 9%–23%) and the US (13%, 95% CI: 6%–22%), while studies from Germany (4%, 95% CI: 2%–6%) and China (2%, 95% CI: 1%–4%) reported relatively lower rates (**Supplementary Figure 2A**). When stratified by study design, the pooled failure rate was 11% (95% CI: 4%–21%) in prospective cohort studies, 9% (95% CI: 5%–15%) in retrospective cohort studies, and 11% (95% CI: 4%–19%) in case series (**Supplementary Figure 2B**). Subgroup analysis by time period confirmed the downward trend, with pooled failure rates of 14.8% (95% CI: 8.7–22.2%) for the early era (2002–2009), 10.2% (95% CI: 6.8–14.2%) for the intermediate era (2011–2015), and 5.6% (95% CI: 3.8–7.8%) for the recent era (2020–2025) (**Supplementary Figure 2C**).

Of the 306 aggregated ductoscopy failure cases, 145 (47.4%) were attributed to the five common clinical categories summarized in **Table 2**. These common clinical categories represent prevalent clinical scenarios rather than specific etiologies. Ductal stenosis was the most common category, accounting for 24.8% of all failures (95% CI: 20.1–30.1%), with a significantly higher proportion than all other common clinical categories (all pairwise comparisons, P < 0.001). The proportions of the remaining common clinical categories were as follows: nipple deformation (11.8%, 95% CI: 8.4–15.9%), no nipple discharge (6.5%, 95% CI: 4.0–9.9%), lesions located within the subareolar main duct (2.9%, 95% CI: 1.4–5.5%), and bloody nipple discharge with no identified lesion (1.3%, 95% CI: 0.4–3.3%). The remaining 161 failure cases (52.6%) were attributed to technical failures (perforation and false passage, 98 cases, 32.0%), device-related issues, procedural pain, impaired visualization, undetermined causes, or other miscellaneous factors (63 cases, 20.6%). Despite this variability, these common clinical categories constitute the major, well-defined clinical challenges in ductoscopic procedures and directly informed the development of the CDOP-PFN classification system described below.

Among the included studies, No.19 (Filipe et al., 2020) demonstrated a markedly high procedural failure rate of 29.1%, significantly exceeding the failure rates of contemporaneous studies (range 3.5%–11.8%). In this study, ductal perforation accounted for 45.2% of all failures, suggesting that the center was experiencing a technical learning plateau during the expansion of indications to include more complex cases. This interpretation is supported by the center’s sequential reports: the initial cohort (2010–2014) reported a failure rate of 13.0%, whereas the later cohort (2015–2017) showed an extrapolated failure rate of 39.8%, reflecting the challenges encountered during the adoption of interventional ductoscopy techniques. To avoid bias from this single outlying center’s experience, a pre-defined objective criterion was established: any study with a failure rate exceeding the mean of contemporaneous studies by more than three standard deviations would be considered an outlier and excluded from the primary time-trend analysis. Study No.19 was the only study meeting this criterion. To assess the impact of this exclusion, we performed a sensitivity analysis by including this study in the time-trend analysis. As shown in **Table 3**, its inclusion raised the recent-era failure rate to 11.4%, attenuated the overall reduction from the early to the recent era to –14.0%, and rendered the trend non-significant (Z = 1.056, P = 0.2909). This marked attenuation underscores the disproportionate influence of this single center’s learning-phase experience on the aggregate trend, reinforcing the appropriateness of its exclusion from the primary analysis. Of note, the overall failure rate across the entire cohort (all 25 studies) was 10.0% (95% CI: 6.8%–13.7%), which is consistent with the meta-analysis estimate.

**Table 3:** Sensitivity analysis: comparison of time-trend results with and without the outlier study.

Analysis	Recent-era failure rate	Overall reduction (early→recent)	Z (trend test)	P value
Primary analysis (excluding Filipe)	6.1%	39.0%	3.285	0.001
Sensitivity analysis (including Filipe)	11.4%	–14.0%	1.056	0.2909

To further evaluate the temporal effects of technological advancement and clinical experience, studies were stratified into three chronological periods based on the distribution of included studies: early era (2002–2009), intermediate era (2011–2015), and recent era (2020–2025). As summarized in **Table 4**, both overall failure rates and technical failures—defined as ductal perforation and entry into a false passage—declined substantially over time. The overall failure rate dropped from 11.1% in the early era to 6.1% in the recent era (39.0% relative reduction), while the technical failure rate decreased from 5.2% to 1.8% (65.4% relative reduction). χ² tests confirmed statistically significant differences across the three periods for both overall failure (χ² = 12.327, df = 2, P = 0.0021) and technical failure (χ² = 14.937, df = 2, P = 0.0006), and the Cochran-Armitage trend tests verified significant linear downward trends for both (Z = 3.285, P = 0.001; and Z = 3.702, P < 0.001, respectively) (**Table 5**). Notably, the proportion of technical failures among all failures decreased from 47.1% (40/85) in the early era to 30.2% (13/43) in the recent era, suggesting that while operator-related technical difficulties have substantially diminished over time with accumulated experience and improved instrumentation, other causes—such as lesion localization challenges, anatomical variations, and patient-related factors—have become relatively more prominent. This shift underscores the necessity of standardized classification systems to address the non-technical complexities encountered in ductoscopic procedures and directly informed the development of the CDOP-PFN framework proposed below.

**Table 4:** Ductoscopy procedures and failures across three time periods (primary analysis, excluding the outlier study).

Time period	Total procedures	Total failures	Failure rate (%)	Technical failures*	Technical failure rate (%)
2002–2009	768	85	11.1	40	5.2
2011–2015	893	111	12.4	32	3.6
2020–2025†	709	43	6.1	13	1.8

*Technical failures are defined as ductal perforation and entry into a false passage.

†Excluding the outlier study (Filipe et al., 2020; failure rate 29.1%).

**Table 5:** Statistical analysis of failure rate trends across time periods.

Test item	Time period	Failure rate (%)	χ² (df)	P value (χ²)	Z (trend test)	P value (trend)
Ductoscopy Failure	2002–2009	11.1	12.327 (2)	0.0021	3.285	0.001
	2011–2015	12.4				
	2020–2025†	6.1				
Technical Failure*	2002–2009	5.2	14.937 (2)	0.0006	3.702	< 0.001
	2011–2015	3.6				
	2020–2025†	1.8				

*Technical failures are defined as ductal perforation and entry into a false passage.

†Excluding the outlier study (Filipe et al., 2020; failure rate 29.1%).

Note: χ² test was used to assess differences in failure rates across the three time periods (df = 2, representing the number of time periods minus 1). The Cochran-Armitage trend test was applied to evaluate the statistical significance of the observed linear downward trend over time. Positive Z-values indicate a decreasing trend in failure rates.

These findings collectively indicate that technological evolution, enhanced visualization systems, standardized procedural approaches, and accumulation of operative experience have significantly improved the safety and effectiveness of ductoscopy. Moreover, they highlight the necessity of structured training programs, competency-based progression, and supervised clinical practice during the initial introduction of ductoscopy to minimize avoidable complications and optimize clinical outcomes.

### 2.4 Association between ductoscopy failure and the malignant breast lesions

Increasing evidence indicates that certain clinical and anatomical features-including ductal stenosis, complete ductal obstruction, absence of spontaneous nipple discharge, and bloody discharge are strongly associated with higher malignant potential in breast lesions. These features may not only complicate endoscopic navigation but also serve as indirect indicators of underlying neoplastic pathology.

Tekin et al. (36) reported two cases in which ductoscopy failed due to complete intraductal occlusion, both subsequently diagnosed as breast cancer (one ductal carcinoma in situ-DCIS with microinvasion and one invasive ductal carcinoma). This observation suggests that failure caused by mechanical obstruction may warrant heightened oncologic suspicion, particularly when consistent with malignant clinical phenotypes.

In another study, among patients without spontaneous nipple discharge, pathological analysis of ductoscopy-derived specimens revealed a markedly elevated breast cancer detection rate of 58% (35/60), comprising both DCIS and invasive carcinoma. Conversely, the detection rate among patients with spontaneous nipple discharge (SND) was significantly lower at 7.5% (3/40) (P < 0.000001) (37). These findings imply that malignant lesions may disrupt or obliterate ductal architecture, thereby reducing or eliminating nipple discharge and complicating clinical recognition.

Similarly, Shen Leihua et al. (38) evaluated 281 patients without palpable nipple discharge (PND) who underwent ductoscopy, identifying intraductal lesions 45.20% of cases, including DCIS in 10.26%. This substantial burden of occult pathology underscores the necessity for comprehensive diagnostic evaluation, even in the absence of classic symptoms.

Yang et al. (32) reported four cases of bloody nipple discharge in which ductoscopy failed to reveal intraductal lesions; however, all were ultimately diagnosed as breast cancer following surgical excision and histopathological examination. These findings reinforce that a negative ductoscopydoes not rule out malignancy, particularly in patients with suspicious bloody discharge, and surgical intervention should be considered to avoid diagnostic delay.

Furthermore, lesions located within the proximal ductal system or immediately posterior to the nipple may hinder endoscopic visualization and advancement, elevating the likelihood of false-negative findings and procedural failure. In the study by Kamali et al. (39), ductoscopy failed in one patient with a large intraductal papilloma that mechanically obstructed ductal entry; postoperative pathology confirmed DCIS, further illustrating the malignant potential of obstructive intraductal lesions.

Taken together, these findings indicate that ductoscopy failure is not solely attributable to operator technique or equipment limitations; rather, it may reflect biologically aggressive disease, ductal architectural distortion, or proximally located obstructive lesions. Building upon reported failure mechanisms (**Figure 2**) and procedural challenges encountered in clinical practice, this study establishes a systematic classification and in-depth analysis of complex ductoscopy cases to support improved diagnostic accuracy and early malignant lesion detection.

## 3 Classification of complex ductoscopy operating procedures

**Figure 5:**
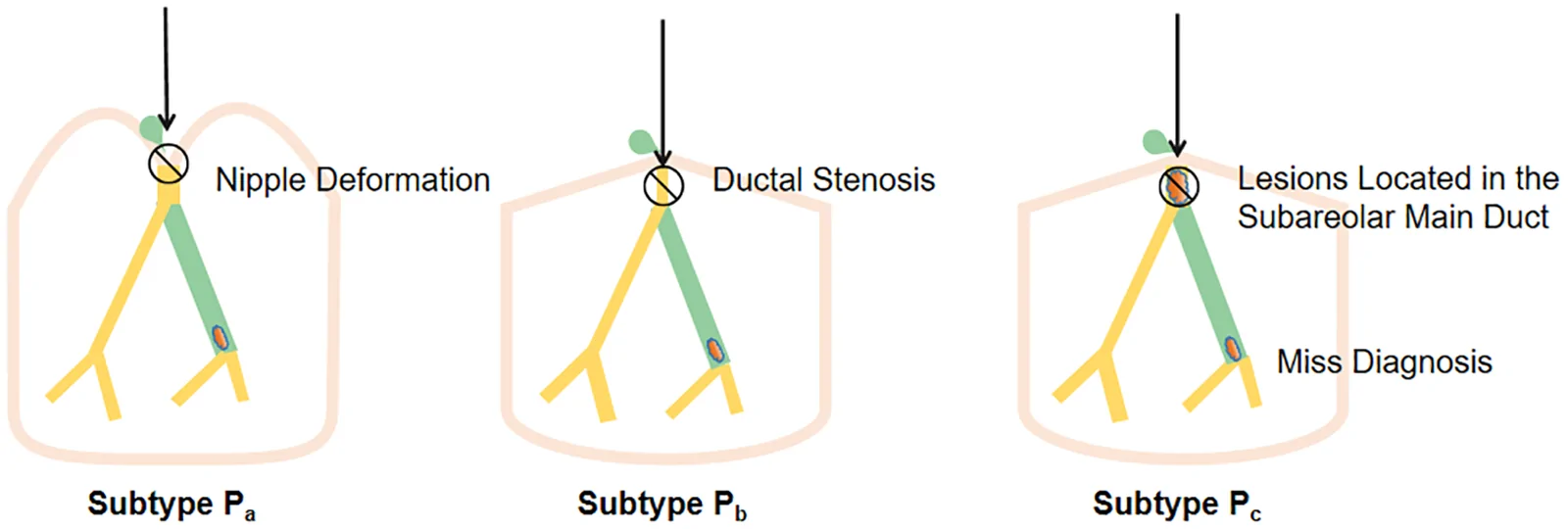
Schematic diagram of type P, CDOP.

The complexity of ductoscopy primarily arises from three fundamental dimensions: (1) Spontaneous nipple discharge present, but the ductoscope fails to enter the target ductal lumen (type P); (2) Bloody nipple discharge present, ductoscopy successfully enters the duct, but no lesion is visualized (type F); (3) No spontaneous nipple discharge, yet breast ultrasonography suggests ductal dilatation and intraductal space-occupying lesions (type N). Drawing upon common intraoperative difficulties encountered in clinical practice, failure causes identified across published literature, and the clinical experience of four senior clinicians from our team specializing in ductoscopy and breast surgery, we developed a standardized classification system for CDOP-PFN into three major types and six subtypes:

Type P: Spontaneous nipple discharge present, but the ductoscope fails to enter the target ductal lumen (**Figure 5**).

- Subtype P_a_: Nipple retraction or structural deformation preventing successful cannulation.
- Subtype P_b_: Ductal stenosis leading to mechanical entry obstruction.
- Subtype P_c_: Lesions located within the proximal/subareolar main duct causing access resistance or lumen occlusion.

**Figure 6:**
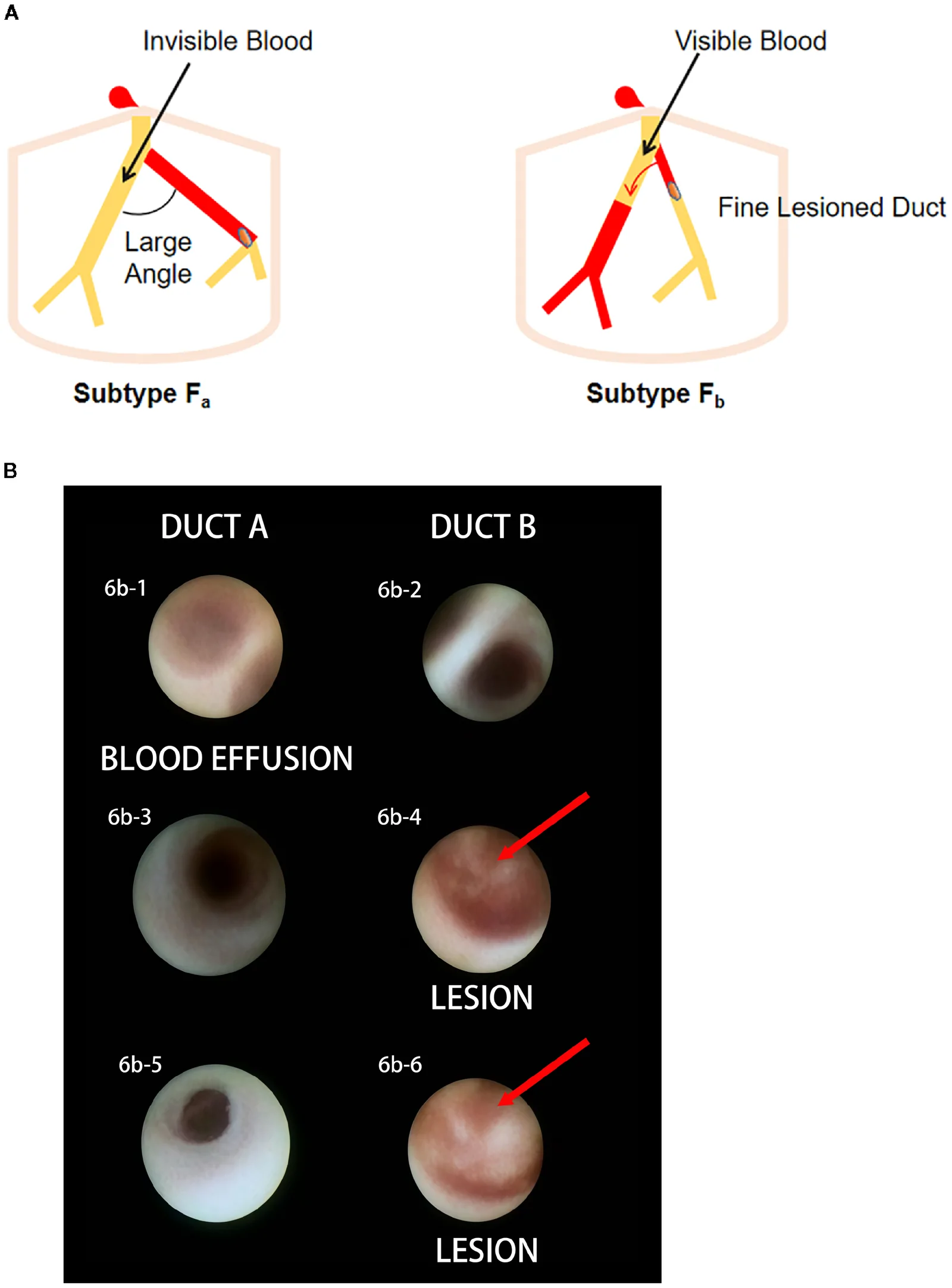
**(A)** Schematic Diagram of Type F, CDOP. **(B)** Representative Intraoperative Ductoscopy Images for Type F_b_, CDOP (Pathology: Breast carcinoma). *Six views from two ductal branches of the same patient. Left column (Duct A): bloody secretions with no visible lesion. Right column (Duct B): bloody secretions with a distinct intraductal lesion at a higher level (arrows). The comparison indicates that the bloody discharge in Duct A originated from the lesion in Duct B via retrograde overflow across the ductal bifurcation. See **Supplementary Video**.

Type F: Bloody nipple discharge present, ductoscopy successfully enters the duct, but no lesion is visualized (**Figure 6**).

- Subtype F_a_: No bloody effusion detected endoscopically; no intraductal lesion visualized.
- Subtype F_b_: Bloody effusion detected endoscopically; but no macroscopic intraductal lesion identified.

**Figure 7:**
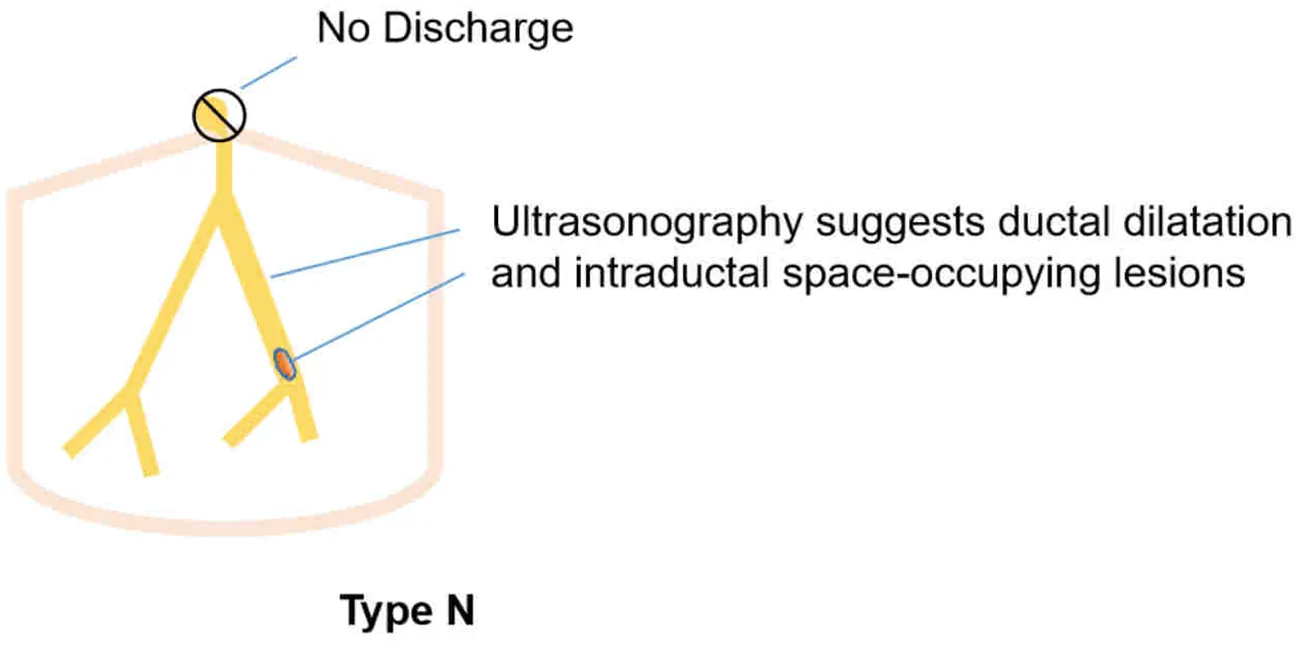
Schematic diagram of type N, CDOP.

Type N: No spontaneous nipple discharge, yet breast ultrasonography suggests ductal dilatation and intraductal space-occupying lesions (**Figure 7**).

## 4 Classification-guided clinical response strategies

**Figure 8:**
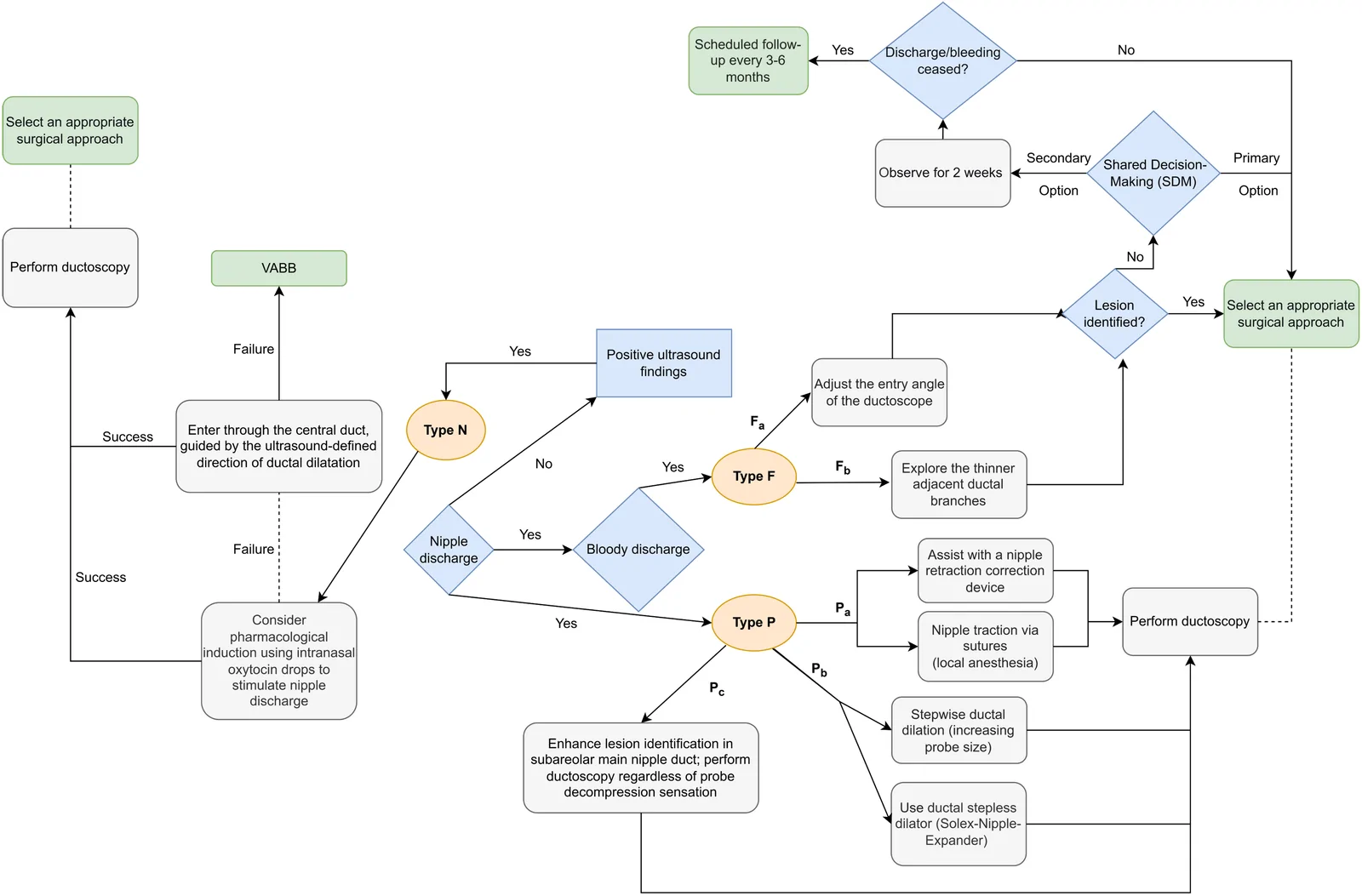
Clinical decision-making flowchart for each CDOP-PFN subtype.

Management strategies for Complex Ductoscopy Operating Procedures (CDOP) are tailored according to the three major types and six subtypes described above (**Figure 8**). The following recommendations, drawn from operative experience and clinical practice, may facilitate ductoscope access, assist in lesion visualization, and possibly reduce false-negative diagnoses.

Type P: Spontaneous nipple discharge present, but the ductoscope fails to enter the target ductal lumen.

- Subtype P_a_: Nipple retraction or structural deformation.

Anatomical distortion of the nipple limits proper ductoscope alignment and cannulation.

Recommended strategies:

- Apply a nipple retraction correction device pre-procedure to restore surface contour.
- During ductoscopy, use suture-assisted nipple traction to optimize ductal axis alignment, improve stability, and facilitate endoscopic entry.

- Subtype P_b_: Ductal stenosis.

Ductal stenosis is often associated with nipple retraction and chronic inflammatory remodeling caused by milk stasis (40).

Recommended strategies:

- Pull out the nipple to straighten the ductal pathway.
- Perform gentle sequential canalization and dilation with a lacrimal probe or stepless ductal dilator (e.g., Solex Nipple Expander).
- Introduce the ductoscope in a stepwise manner once patency is restored.

- Subtype P_c_: Lesions located within the proximal/subareolar main duct.

Proximal lesions may obstruct cannulation and increase the risk of missed diagnosis.

Recommended strategies:

- Attempt careful endoscopic advancement even if mild resistance is encountered.
- Repeat ultrasound evaluation to reassess subareolar ductal lesions.
- If ductoscopy remains unsuccessful but imaging strongly suggests intraductal lesions, proceed directly to surgical excision to avoid diagnostic delay.

Type F: Bloody nipple discharge present, ductoscopy successfully enters the duct, but no lesion is visualized.

- Subtype F_a_: No bloody secretions or visible lesions endoscopically.

Sharp angulation between the critically short main duct and pathological side branch may hinder navigation.

Recommended strategies:

- Adjust the endoscope’s orientation and angle.
- Modify entry direction dynamically to increase the likelihood of entering the affected branch duct.

- Subtype F_b_: Bloody secretions present, but no lesions endoscopically.

Proximal lesions in the thinner duct may cause blood to reflux into adjacent wider ducts, obscuring true lesion location.

Recommended strategies:

- Focus exploration on thinner adjacent ductal branches near the blood accumulation point.
- Prioritize examining upstream narrow ducts rather than only following the main ductal pathway.

Type N: No spontaneous nipple discharge, and discharge cannot be induced by physical maneuvers (e.g., pressing or nipple aspiration), yet breast ultrasonography suggests ductal dilatation and intraductal space-occupying lesions.

Absence of spontaneous nipple discharge is not an absolute contraindication for ductoscopy. For patients in this category, a stepwise approach is recommended:

Recommended strategies:

- Consider pharmacological induction using intranasal oxytocin (41) drops to stimulate nipple discharge, which may facilitate ductal access and targeted cannulation in selected patients without spontaneous discharge. The induced nipple aspirate fluid (NAF) can be used for duct mapping and to guide the endoscope toward the target duct (37).
- If induction fails or is not feasible, attempt ductoscopy by entering through the central duct, with the direction of advancement guided by the ultrasonographically defined orientation of ductal dilatation (42).
- If ductoscopy remains unsuccessful despite the above measures, or if an intraductal lesion is suspected but cannot be adequately visualized or biopsied endoscopically, proceed to ultrasound-guided vacuum-assisted breast biopsy(VABB).

### 4.1 Additional considerations

For patients at high risk for breast cancer, particularly those with bloody or serous discharge, even a negative ductoscopic examination warrants vigilance. However, the decision to proceed with surgical intervention should not be based on ductoscopy findings alone. Instead, it should be individualized, taking into account BI-RADS category, MRI findings, cytology results, patient risk factors, and shared decision-making.

Suggested next steps:

- Re-evaluate surgical indications based on comprehensive imaging and clinical risk assessment.
- Consider microductectomy with methylene blue duct mapping to localize suspicious ducts and improve lesion capture accuracy (43), which is especially valuable as a secondary diagnostic pathway following unsuccessful ductoscopy.

## 5 Conclusions and prospects

This systematic review and meta-analysis quantified the overall ductoscopy failure rate at 10.0% (95% CI: 6.8%–13.7%; 95% PI: 0.0%–31.4%), revealing substantial heterogeneity (I² = 87%) across studies. Based on this evidence and extensive clinical experience, for the first time, this study proposes a structured three-type, six-subtype classification framework for Complex Ductoscopy Operating Procedures (CDOP-PFN). This system clarifies the principal clinical scenarios and mechanistic factors associated with challenging ductoscopic procedures. Based upon this framework, we established classification-guided, stepwise clinical response strategy. This would standardize procedural decision-making, reduce operator-dependent variability, and enhance the accuracy and safety of intraductal lesion diagnosis and management.

The study has several limitations. The high heterogeneity in the meta-analysis limits the generalizability of the pooled estimate. The CDOP-PFN classification, while derived from literature and accumulated clinical expertise, requires prospective, multicenter validation to confirm its clinical utility and reliability. Additionally, the analysis depends on reported data, which may be subject to publication and reporting biases.

Future efforts should focus on validating the CDOP-PFN framework in prospective studies. Improving ductoscopy success rates requires not only accurate recognition of complex procedural patterns and mastery of standardized management pathways, but also ongoing advancements in procedural support technologies. In the future, two key innovations hold particular promise for accelerating the evolution of ductoscopy: (i) simulation-based virtual training platforms - enabling standardized skill acquisition, hands-on simulation of ductal anatomy, and shortened learning curves for novice operators; (ii) Artificial intelligence-enhanced ductoscopic imaging systems — integrating automated lesion detection, blood-tracking algorithms, and real-time decision support to enhance diagnostic precision and reduce missed lesions.

The development and clinical adoption of these technologies will offer strong technical support for minimally invasive, image-guided management of breast diseases. They are expected to accelerate the transition toward precision endoscopy, intelligent diagnostic assistance, and standardized micro-interventional treatment in breast surgery.

Beyond procedural success, long-term follow-up outcomes after ductoscopy-assisted papilloma excision represent an important area for future investigation. While the present review focuses on procedural failure mechanisms, the clinical relevance of ductoscopy ultimately depends on whether improved lesion localization translates into durable symptom resolution and low recurrence rates. Emerging evidence from single-center series suggests favorable short-term outcomes, but well-designed prospective studies with standardized follow-up protocols are needed to establish the long-term effectiveness of ductoscopy-guided excision. This represents a key priority for our ongoing research program.

## Data Availability

The original contributions presented in the study are included in the article/**Supplementary Material**, further inquiries can be directed to the corresponding author/s.

## Supplementary material

Supplementary File 1 Quality Assessment of Included Studies. The methodological quality of the 33 included articles was assessed using design-specific tools: NOS for cohort studies, JBI for case series, QUADAS-2 for diagnostic studies, and RoB 2 for RCTs. Of the 33 articles, 32 (97.0%) were rated Good and 1 (3.0%) Fair; none were Poor. All five case series assessed by JBI were rated Good; the diagnostic study (Grunwald et al., 2006) showed low risk of bias on QUADAS-2; and the single RCT (Gui et al., 2021) was rated low risk on all RoB 2 domains. After merging overlapping cohorts into 25 independent groups, 24 (96%) remained Good and 1 (4%) Fair. The sole Fair-rated study (Zielinski et al., 2015; NOS 6/9) was retained, as sensitivity analysis excluding it did not materially change the pooled estimate. The predominance of Good-rated studies across all tools supports the credibility of the findings.

Supplementary File 2 Subgroup Analyses of Ductoscopy Failure Rates. This file contains the detailed results of subgroup meta-analyses performed to explore potential sources of heterogeneity in the primary analysis (I² = 87.1%). The file includes: **Supplementary Figures 2A–C** — Forest plots of subgroup analyses stratified by geographic region (S2A), study design (S2B), and time period (S2C). Summary Table — A summary of pooled failure rates with 95% confidence intervals and I² statistics for each subgroup. These subgroup analyses support the robustness of the overall pooled estimate and provide insights into the variability of ductoscopy failure rates across different clinical settings.

Supplementary Video The video illustrates the CDOP F_b_ subtype, in which the lesion is situated relatively high within the mammary duct. This anatomical location leads to blood overflow into adjacent ductal branches following lesion bleeding. Clinically, this may present as the detection of bloody discharge without identification of the corresponding lesion under ductoscopy. Other subtypes are also associated with clinically complex scenarios; however, due to the challenges in preserving complete video documentation, they will not be elaborated on in detail herein.
