# Supplementary material for: Research progress on the classification and clinical response strategies for “complex ductoscopy operating procedure”
Source: Front Oncol. 2026 Aug 11;16:1794764. doi: 10.3389/fonc.2026.1794764 (PMC13503262; doi:10.3389/fonc.2026.1794764)
Supplement: Supplementary file 1 [file Table1.docx]

**Supplementary File S1: Quality Assessment of Included Studies**

**Table S1. Summary of All Reviewed Articles with Study Design, Assessment Tool,**

**and Final Inclusion Status (N=33)**

| **No.** | **Study** | **N** | **Design** | **Tool** | **Status** |
| --- | --- | --- | --- | --- | --- |
| 1 | Dooley, 2002 | 27 | Prospective Cohort | NOS | Current #1 |
| 2 | Dietz JR et al, 2002 | 119 | Retrospective Cohort | NOS | Merged -> #2 |
| 3 | Sharma R et al, 2005 | 235 | Retrospective Cohort | NOS | Merged -> #2 |
| 4 | Kim et al, 2004 | 30 | Prospective Cohort | NOS | Current #3 |
| 5 | Moncrief et al, 2005 | 68 | Retrospective Cohort | NOS | Current #4 |
| 6 | Beechey-Newman, 2005 | 50 | Retrospective Cohort | NOS | Current #5 |
| 7 | Hünerbein M et al, 2006 | 54 | Prospective Cohort | NOS | Merged -> #6 |
| 8 | Hünerbein M et al, 2006 | 38 | Prospective Cohort | NOS | Merged -> #6 |
| 9 | Dubowy A et al, 2011 | 102 | Prospective Cohort | NOS | Merged -> #6 |
| 10 | Al Sarakbi et al, 2006 | 26 | Case Series | JBI | Current #7 |
| 11 | Grunwald et al, 2006 | 20 | Diagnostic Study | QUADAS-2 | Current #8 |
| 12 | Tondre et al, 2008 | 308 | Prospective Cohort | NOS | Current #9 |
| 13 | Bender et al, 2009 | 126 | Retrospective Cohort | NOS | Merged -> #15 |
| 14 | Simpson et al, 2009 | 66 | Case Series | JBI | Current #10 |
| 15 | Cyr et al, 2011 | 25 | Prospective Cohort | NOS | Current #11 |
| 16 | Fisher et al, 2011 | 121 | Retrospective Cohort | NOS | Current #12 |
| 17 | Khan et al, 2011 | 59 | Prospective Cohort | NOS | Current #13 |
| 18 | Albrecht et al, 2013 | 100 | Retrospective Cohort | NOS | Current #14 |
| 19 | Kamali et al, 2010 | 236 | Case Series | JBI | Merged -> #15 |
| 20 | Kamali et al, 2014 | 456 | Case Series | JBI | Merged -> #15 |
| 21 | Zielinski et al, 2015 | 164 | Retrospective Cohort | NOS | Current #16 |
| 22 | Liu M et al, 2015 | 238 | Prospective Cohort | NOS | Current #17 |
| 23 | Ohlinger et al, 2014 | 214 | Prospective Cohort | NOS | Merged -> #18 |
| 24 | Ohlinger et al, 2020 | 224 | Prospective Cohort | NOS | Merged -> #18 |
| 25 | Waaijer et al, 2015 | 82 | Prospective Cohort | NOS | Merged -> #19 |
| 26 | Filipe et al, 2017 | 149 | Retrospective Cohort | NOS | Merged -> #19 |
| 27 | Filipe et al, 2020 | 213 | Retrospective Cohort | NOS | Merged -> #19 |
| 28 | Chang et al, 2020 | 223 | Retrospective Cohort | NOS | Current #20 |
| 29 | Gui et al, 2021 | 34 | RCT | RoB 2 | Current #21 |
| 30 | Yang et al, 2023 | 112 | Retrospective Cohort | NOS | Current #22 |
| 31 | Makineli S et al, 2024 | 35 | Retrospective Cohort | NOS | Current #23 |
| 32 | Makineli S et al, 2025 | 24 | Phase II Trial | JBI | Current #24 |
| 33 | Cabioglu et al, 2025 | 57 | Retrospective Cohort | NOS | Current #25 |

Note: 33 individual articles.

*“Current #X” indicates the study was retained as an independent dataset in the meta-analysis and corresponds to the numbering in Table 1 of the main manuscript. “Merged -> #X” indicates the study was merged with another report from the same cohort; the merged dataset corresponds to the numbering in Table 1.

**Table S2a. NOS for Cohort Studies (N=26)**

| **No.** | **Study** | **Design** | **Selection (Max 4)** | **Comparability (Max 2)** | **Outcome (Max 3)** | **Total (Max 9)** | **Quality** |  |
| --- | --- | --- | --- | --- | --- | --- | --- | --- |
| 1 | Dooley, 2002 | Prosp. Cohort | 3 | 1 | 3 | 7 | Good |  |
| 2 | Dietz JR et al, 2002 | Retro. Cohort | 3 | 1 | 3 | 7 | Good |  |
| 3 | Sharma R et al, 2005 | Retro. Cohort | 3 | 1 | 3 | 7 | Good |  |
| 4 | Kim et al, 2004 | Prosp. Cohort | 3 | 2 | 3 | 8 | Good |  |
| 5 | Moncrief et al, 2005 | Retro. Cohort | 3 | 2 | 3 | 8 | Good |  |
| 6 | Beechey-Newman, 2005 | Retro. Cohort | 3 | 2 | 3 | 8 | Good |  |
| 7 | Hünerbein M et al, 2006 | Prosp. Cohort | 3 | 2 | 3 | 8 | Good |  |
| 8 | Hünerbein M et al, 2006 | Prosp. Cohort | 3 | 2 | 3 | 8 | Good |  |
| 9 | Dubowy A et al, 2011 | Prosp. Cohort | 3 | 2 | 3 | 8 | Good |  |
| 10 | Tondre et al, 2008 | Prosp. Cohort | 4 | 2 | 3 | 9 | Good |  |
| 11 | Bender et al, 2009 | Retro. Cohort | 3 | 2 | 3 | 8 | Good |  |
| 12 | Cyr et al, 2011 | Prosp. Cohort | 4 | 2 | 3 | 9 | Good |  |
| 13 | Fisher et al, 2011 | Retro. Cohort | 4 | 2 | 3 | 9 | Good |  |
| 14 | Khan et al, 2011 | Prosp. Cohort | 4 | 2 | 3 | 9 | Good |  |
| 15 | Albrecht et al, 2013 | Retro. Cohort | 3 | 2 | 3 | 8 | Good |  |
| 16 | Zielinski et al, 2015 | Retro. Cohort | 3 | 1 | 2 | 6 | Fair | Lacked comparator group; did not adjust for confounders; high dropout (41.5%) |
| 17 | Liu M et al, 2015 | Prosp. Cohort | 4 | 2 | 3 | 9 | Good |  |
| 18 | Ohlinger et al, 2014 | Prosp. Cohort | 4 | 2 | 3 | 9 | Good |  |
| 19 | Ohlinger et al, 2020 | Prosp. Cohort | 4 | 2 | 3 | 9 | Good |  |
| 20 | Waaijer et al, 2015 | Prosp. Cohort | 4 | 2 | 3 | 9 | Good |  |
| 21 | Filipe et al, 2017 | Retro. Cohort | 4 | 2 | 3 | 9 | Good |  |
| 22 | Filipe et al, 2020 | Retro. Cohort | 4 | 2 | 3 | 9 | Good |  |
| 23 | Chang et al, 2020 | Retro. Cohort | 3 | 2 | 3 | 8 | Good |  |
| 24 | Yang et al, 2023 | Retro. Cohort | 4 | 2 | 3 | 9 | Good |  |
| 25 | Makineli S et al, 2024 | Retro. Cohort | 3 | 2 | 3 | 8 | Good |  |
| 26 | Cabioglu et al, 2025 | Retro. Cohort | 3 | 2 | 3 | 8 | Good |  |

**Table S2b. JBI Checklist for Case Series (N=5)**

| **No.** | **Study** | **N** | **Q1** | **Q2** | **Q3** | **Q4** | **Q5** | **Q6** | **Q7** | **Q8** | **Q9** | **Q10** | **Score** | **Quality** |
| --- | --- | --- | --- | --- | --- | --- | --- | --- | --- | --- | --- | --- | --- | --- |
| 10 | Al Sarakbi 2006 | 26 | Y | Y | Y | N | Y | Y | Y | Y | Y | Y | 9/10 | Good |
| 14 | Simpson 2009 | 66 | Y | Y | Y | Y | Y | Y | Y | Y | Y | Y | 10/10 | Good |
| 19 | Kamali 2010 | 236 | Y | Y | Y | Y | Y | Y | Y | Y | Y | Y | 10/10 | Good |
| 20 | Kamali 2014 | 456 | Y | Y | Y | Y | Y | Y | Y | Y | Y | Y | 10/10 | Good |
| 32 | Makineli S et al, 2025 | 24 | Y | Y | Y | Y | N | Y | Y | Y | Y | Y | 9/10 | Good |

**Table S2c. QUADAS-2 — Grunwald et al, 2006 (n=20)**

| Grunwald 2006 | **Domain** | **Risk of Bias** | **Applicability** | **Evidence** |
| --- | --- | --- | --- | --- |
|  | Patient Selection | Low | Low | Prospective consecutive; standard imaging |
|  | Index Test (Ductoscopy) | Low | Low | Karl Storz 1.3mm; standardized |
|  | Reference (Histopathology) | Low | Low | Open biopsy; gold standard |
|  | Flow and Timing | Unclear | Low | Both tests; interval <2w; small n=20. Unclear if all patients received the same reference standard |

**Table S2d. Cochrane RoB 2 — Gui et al, 2021 (RCT, n=34)**

| Gui et al, 2021 (INTEND 1 RCT) | **Domain** | **Risk of Bias** | **Evidence** |
| --- | --- | --- | --- |
|  | D1: Randomization process | Low | Computer-generated; sealed envelope |
|  | D2: Deviations from interventions | Low | No cross-over; modified ITT |
|  | D3: Missing outcome data | Low | All 34 patients completed follow-up |
|  | D4: Measurement of outcome | Low | Margin status by histopathology |
|  | D5: Selection of reported result | Low | Pre-registered (CCR2735) |
|  | **Overall** | **Low Risk of Bias** | **Quality: Good. All 5 domains Low Risk.** |

**Quality Assessment Conclusion**

Of the 33 articles reviewed, 32 (97.0%) were rated Good quality and 1 (3.0%) as Fair. No studies were classified as Poor. The only Fair-rated study: Zielinski et al, 2015 (NOS score 6/9) . Reason for Fair rating:

Comparability (1/2): The study did not adequately control for confounding. It lacked a comparator group and did not adjust for age, discharge type, or prior breast surgery.

Outcome (2/3): Follow-up was incomplete. The paper reports that “in the group of patients disqualified from surgical examination, the procedure was abandoned in 51 (41.5%) patients.” This high dropout rate limits outcome verification. Follow-up duration was also variable.

**Quality by tool: NOS: 25 Good, 1 Fair | JBI: 5/5 Good | QUADAS-2: Low Risk | RoB 2: Low Risk**

**Merged into 25 analysis groups: 24 Good (96%), 1 Fair (4%)**

Despite the Fair rating, Zielinski et al provides useful 5-year data (2004–2010) on ductoscopy failure and is included in the meta-analysis. A sensitivity analysis excluding this study did not materially alter the pooled effect estimate.
