# Supplementary material for: Research progress on the classification and clinical response strategies for “complex ductoscopy operating procedure”
Source: Front Oncol. 2026 Aug 11;16:1794764. doi: 10.3389/fonc.2026.1794764 (PMC13503262; doi:10.3389/fonc.2026.1794764)
Supplement: Supplementary file 2 [file Table2.docx]

Supplementary File S2: Meta-Analysis of the Overall Failure Rate of Ductoscopy

Corresponding to:

“Systematic Review on Complex Ductoscopy Classification and Clinical Strategies” (Manuscript)

Methods

A meta-analysis was performed to pool the overall failure rate of ductoscopy from the 22 studies included in the systematic review. Owing to the anticipated heterogeneity across studies, a random-effects model (DerSimonian--Laird method) was employed for data synthesis. Heterogeneity among studies was quantified using the I² statistic, with I² values of 25%, 50%, and 75% conventionally indicating low, moderate, and high heterogeneity, respectively.

Results

Summary of Pooled Effect
The pooled failure rate was 11.4% (95% CI: 8.8% -- 14.3%).
Heterogeneity: I² = 87% (P < 0.001), indicating substantial heterogeneity across the included studies.

Table S2-1. Failure rates of ductoscopy from included studies and pooled estimate

| Study  (Author, Year) | Total Procedures | Failure Cases | Failure Rate  (95% CI) |
| --- | --- | --- | --- |
| Dietz et al, 2002 | 121 | 14 | 11.6% (6.5–18.6%) |
| Kim et al, 2004 | 30 | 11 | 36.7% (20.0–56.0%) |
| Moncrief et al, 2005 | 68 | 9 | 13.2% (6.2–23.6%) |
| Hünerbein et al, 2006 | 54 | 4 | 7.4% (2.1–17.9%) |
| Al Sarakbi et al, 2006 | 13 | 3 | 23.1% (5.0–53.8%) |
| Grunwald et al, 2006 | 20 | 3 | 15.0% (3.2–37.9%) |
| Tondre et al, 2008 | 216 | 28 | 13.0% (8.9–17.9%) |
| Bender et al, 2009 | 126 | 24 | 19.0% (12.7–26.9%) |
| Simpson et al, 2009 | 66 | 3 | 4.5% (0.9–12.7%) |
| Kamali et al, 2010 | 249 | 43 | 17.2% (12.8–22.5%) |
| Cyr et al, 2011 | 27 | 9 | 33.3% (16.6–54.3%) |
| Fisher et al, 2011 | 121 | 2 | 1.6% (0.2–5.9%) |
| Khan et al, 2011 | 59 | 9 | 15.3% (7.2–26.8%) |
| Albrecht et al, 2013 | 100 | 2 | 2.0% (0.2–7.0%) |
| Zielinski et al, 2015 | 164 | 16 | 9.8% (5.7–15.3%) |
| Liu et al, 2015 | 238 | 5 | 2.1% (0.7–4.9%) |
| Ohlinger et al, 2020 | 224 | 10 | 4.5% (2.2–8.1%) |
| Filipe et al, 2020 | 213 | 62 | 29.1% (23.3–35.5%) |
| Chang et al, 2020 | 223 | 13 | 5.8% (3.1–9.8%) |
| Yang et al, 2023 | 112 | 4 | 3.6% (1.0–9.0%) |
| Makineli et al, 2024 | 35 | 7 | 20.0% (8.4–37.7%) |
| Makineli et al, 2024* | 24 | 3 | 12.5% (2.7–32.4%) |
| Pooled Estimate  (Random-Effects Model) | 2,503 | 284 | 11.4% (8.8–14.3%) |

Notes: The two studies by Makineli et al (2024) represent distinct cohorts from the same institution.
CI, confidence interval.

Heterogeneity Analysis and Interpretation

The meta-analysis revealed substantial heterogeneity across the included studies (I² = 87%, P < 0.001), which is likely attributable to several factors:
• Procedural era and technological evolution: Advances in ductoscopy technology over the study period (2002–2024) may have improved procedural success rates, leading to differences in failure rates between earlier and later studies.
• Operator expertise: Differences in operator proficiency and experience across institutions could contribute to variability in failure rates.
• Patient selection criteria: Differences in patient populations (e.g., age, disease severity, comorbidities) across studies may influence the rate of procedural failure.

**Subgroup Analyses of Ductoscopy Failure Rates**

To explore the potential sources of the substantial heterogeneity observed in the primary meta-analysis (I² = 87.1%), we performed pre-specified subgroup analyses stratified by geographic region, study design, and time period. All analyses were conducted using a random-effects model with the Freeman-Tukey double arcsine transformation and restricted maximum likelihood estimation (REML). The results of these subgroup analyses are presented in Figures S2A–S2C.

## Supplementary Figure S2A. Subgroup Analysis by Geographic Region

*Figure S2A. Subgroup meta-analysis of ductoscopy failure rates stratified by geographic region. Studies were grouped according to the country in which the study was conducted: USA, UK, Germany, Canada, Turkey, Poland, China, Netherlands, and Hong Kong. The size of each square is proportional to the study weight (inverse variance). Horizontal lines represent 95% confidence intervals (CIs). Diamonds represent pooled estimates for each subgroup with their 95% CIs. Heterogeneity within each subgroup is shown as I² values. The analysis shows substantial geographic variation in ductoscopy failure rates, with the Netherlands reporting the highest pooled failure rate (23%, 95% CI: 14%–33%) and China reporting the lowest (2%, 95% CI: 1%–4%).*

## Supplementary Figure S2B. Subgroup Analysis by Study Design

*Figure S2B. Subgroup meta-analysis of ductoscopy failure rates stratified by study design. Studies were categorized as prospective cohort, retrospective cohort, case series, diagnostic study, randomized controlled trial (RCT), or phase II trial. The size of each square is proportional to the study weight (inverse variance). Horizontal lines represent 95% confidence intervals (CIs). Diamonds represent pooled estimates for each subgroup with their 95% CIs. Heterogeneity within each subgroup is shown as I² values. The analysis shows that the pooled failure rates were comparable across the three main study design categories: prospective cohort studies (11%, 95% CI: 4%–21%), retrospective cohort studies (9%, 95% CI: 5%–15%), and case series (11%, 95% CI: 4%–19%). Test for subgroup differences: χ² = 0.50, df = 6, P = 0.032.*

## Supplementary Figure S2C. Subgroup Analysis by Time Period

*Figure S2C. Subgroup meta-analysis of ductoscopy failure rates stratified by three time periods based on the distribution of included studies: early era (2002–2009), intermediate era (2011–2015), and recent era (2020–2025). The outlier study (Filipe et al., 2020) was excluded from this analysis as per the pre-specified criterion (see Section 2.3 of the main manuscript). The size of each square is proportional to the study weight (inverse variance). Horizontal lines represent 95% confidence intervals (CIs). Diamonds represent pooled estimates for each subgroup with their 95% CIs. The analysis confirms a progressive decline in failure rates from the early era (14.8%, 95% CI: 8.7%–22.2%) to the intermediate era (10.2%, 95% CI: 6.8%–14.2%) and the recent era (5.6%, 95% CI: 3.8%–7.8%), supporting the downward trend observed in the descriptive time-trend analysis (Tables 3.1–3.2 of the main manuscript).*

## Summary of Subgroup Analyses

| **Subgroup** | **Categories** | **Studies, n** | **Pooled Failure Rate** | **95% CI** | **I²** |
| --- | --- | --- | --- | --- | --- |
| **Geographic Region** | USA | 7 | 13% | 6%–22% | 84.2% |
|  | UK | 3 | 15% | 9%–23% | 0% |
|  | Germany | 4 | 4% | 2%–6% | 25.9% |
|  | Netherlands | 3 | 23% | 14%–33% | 48.3% |
|  | China | 2 | 2% | 1%–4% | 0% |
|  | Turkey | 2 | 9% | 1%–24% | 88.3% |
|  | Canada | 1 | 5% | — | — |
|  | Poland | 1 | 10% | — | — |
|  | Hong Kong | 1 | 29% | — | — |
| **Study Design** | Prospective Cohort | 8 | 11% | 4%–21% | 87.3% |
|  | Retrospective Cohort | 11 | 9% | 5%–15% | 90.3% |
|  | Case Series | 3 | 11% | 4%–19% | 74.3% |
| **Time Period** | 2002–2009 | 10 | 14.8% | 8.7%–22.2% | — |
|  | 2011–2015 | 7 | 10.2% | 6.8%–14.2% | — |
|  | 2020–2025 | 7 | 5.6% | 3.8%–7.8% | — |

*Note: All pooled estimates were calculated using a random-effects model with Freeman-Tukey double arcsine transformation and REML estimation. CI = confidence interval; I² = heterogeneity statistic; — = not applicable (single study or insufficient data for I² calculation).*
